# Smart Electric Vehicle Charging Management Using Reinforcement Learning on FPGA Platforms

**DOI:** 10.3390/s25082585

**Published:** 2025-04-19

**Authors:** Udhaya Mugil Damodarin, Gian Carlo Cardarilli, Luca Di Nunzio, Marco Re, Sergio Spanò

**Affiliations:** Department of Electronic Engineering, Tor Vergata University of Rome, Via del Politecnico 1, 00133 Rome, Italy

**Keywords:** sensors data processing, reinforcement learning, Q-learning, FPGA, hardware acceleration, green mobility, electric vehicles, sustainability, embedded systems

## Abstract

This paper presents a smart electric vehicle (EV) charging management system that integrates Reinforcement Learning intelligence on a Field-Programmable Gate Array (FPGA) platform. The system is based on the Q-learning algorithm, where the RL agent perceives environmental conditions, captured through hardware sensors such as current, voltage, and priority indicators, and makes optimal charging decisions to address grid stress and prioritize charging needs. The FPGA implementation leverages hardware design strategies to ensure efficient operation and real-time response within a limited amount of required energy, allowing for its implementation in embedded applications and possibly enabling the use of an energy harvesting power source, like a small solar panel. The proposed design effectively manages multiple EV chargers by dynamically allocating current and prioritizing charging tasks to maintain service quality. Through intelligent decision making, informed by continuous sensor feedback, the system adapts to fluctuating grid conditions and optimizes energy distribution. Key findings highlight the system’s ability to maintain stable operation under varying demand conditions, improving power efficiency, safety, and service reliability. Moreover, the design is scalable, enabling seamless expansion for larger installations by following consistent architectural guidelines. This FPGA-based solution combines RL intelligence, sensor-based environmental perception, and robust hardware design, offering a practical framework for an efficient EV charging infrastructure in modern smart grid environments.

## 1. Introduction

The growing adoption for electric vehicles (EVs) worldwide is driving an unprecedented demand for an efficient and intelligent charging infrastructure. As electric vehicles become more commonplace, the load they place on electrical grids is rapidly increasing; in residential areas alone, charging EVs could increase peak electricity demand by up to 30% [1]. Public charging installations are expanding, with global charging points rising by about 40% in 2023, yet this growth must accelerate further to keep up with projected EV uptake [2]. Crucially, charging networks must scale in a smart way to ensure that the rising electricity demand from EVs does not over-stretch the power grids [3,4]. This context has created an urgent need for advanced management strategies that can make the charging infrastructure more intelligent, adaptive, and capable of handling the coming influx of electric vehicles.

Current EV charging practices face several key challenges. Grid stress is a primary concern, as uncoordinated high-power charging of many vehicles can lead to load spikes, transformer overheating, and voltage deviations in the distribution network. Utilities warn that, without smart control, surges in electric vehicle charging during peak hours could push local networks beyond safe limits, risking reliability and stability [5,6]. Furthermore, the energy distribution in today’s charging systems is often inefficient, since most charging occurs first on a first come, first served basis or follows simplistic rules, failing to optimally balance the load. Typically, there is a lack of prioritization mechanisms to decide which vehicles should be parked when capacity is limited, leading to poor use of the available power and longer waiting times during congestion [7]. For example, without smart scheduling, all electric vehicles could start charging as soon as they are plugged in, although postponing charging of certain vehicles could potentially minimize peak load or capitalize on reduced tariffs. These limitations underscore the need for dynamic real-time control, as an effective charging management system must respond instantaneously to fluctuations in grid conditions and to the arrival or departure of vehicles. In sum, managing EV charging is a complex multi-factor decision problem, a challenge that static algorithms and preset heuristics find difficult to address, particularly as the size grows [8].

This is possible thanks to the benefits of rich sensor data, supporting the ability to adapt to dynamic grid and vehicle states. EV charging occurs in a complex environment where both the grid supply and the battery’s condition can fluctuate. Grid-side sensors (monitoring feeder voltage, frequency, or transformer loading) inform the management system of the external power conditions, allowing it to modulate the EV’s charging rate to support grid stability. For example, if sensors detect a drop in line voltage or frequency (indicating a stressed grid), the controller can temporarily taper the charging power to avoid exacerbating the issue, then resume full charging when conditions normalize. This adaptive behavior is crucial as uncontrolled charging of many EVs can cause voltage fluctuations and reliability problems in the local network [9,10]. By continuously sensing grid parameters, the system can mitigate such impacts, essentially using the EVs as flexible loads that respond to real-time measurements.

In this context, Reinforcement Learning (RL) [11] offers a promising approach to intelligently manage EV charging [12,13]. RL algorithms excel at decision making in complex and uncertain environments by learning optimal policies through trial-and-error interaction. Rather than relying on a fixed schedule or model, an RL-based controller can adapt its charging decisions based on feedback from the grid and vehicles, improving performance over time. Notably, RL agents can handle the kind of real-time, adaptive control that EV charging requires: they observe the current state (e.g., grid load, available generation, battery levels of connected EVs) and choose charging actions that are refined by continuous learning. Previous studies highlight that such agents can adapt to real-time data and evolving conditions in dynamic systems such as EV charging networks [14,15,16,17,18].

Although RL can provide the “brain” for smart charging, deploying this intelligence in a real-world infrastructure requires a suitable computing platform. Field-Programmable Gate Arrays (FPGAs) offer significant advantages for implementing RL-based control in embedded systems such as charging stations. FPGAs are reconfigurable hardware devices that are known for their energy efficiency and speed when executing specialized algorithms. An FPGA implementation can exploit parallelism and custom datapaths to accelerate the computations involved in RL (e.g., state evaluations, Q-value updates), achieving high throughput with minimal latency. Importantly, this performance comes with low power consumption; an optimized RL controller on FPGA hardware can run with a much smaller energy footprint than a general-purpose processor or even a GPU (Graphics Processing Unit) [19]. For example, previous works on hardware Q-learning demonstrated that a carefully designed FPGA accelerator used fewer resources, ran faster, and dissipated less power than equivalent CPU (Central Processing Unit) based solutions [20,21]. These characteristics make FPGAs ideal for embedded deployment, as the RL logic can be integrated directly into a charging station or grid-edge device, operating in real time without drawing substantial power from the system. Furthermore, the deterministic execution and reliability of FPGAs align well with the needs of power system applications, which require consistent quick responses. Implementing the intelligent charging manager on an FPGA yields a solution that is not only adaptive and smart but also hardware efficient, a crucial factor for a sustainable wide-scale rollout of smart chargers.

Running the RL agent on an FPGA means that sensor data can be ingested and processed with ultra-low latency and high parallelism. Unlike a conventional software controller, an FPGA can read from dozens of sensors simultaneously and update the charging control output within microseconds or nanoseconds, which is ideal for the fast dynamics of power electronics. This inherent parallelism and speed of FPGAs align well with the input from multiple sensors and the real-time requirements of smart charging [22]. In practical terms, the RL agent on FPGA can continuously stream in measurements and compute optimal control actions on the fly, without the delays of an OS or network. This tightly coupled sensing-and-actuation loop results in more responsive and stable charging control.

The synergy between FPGA and sensors also facilitates integrating new sensor modalities, extending the RL agent’s perceptive range. It can be stated that implementing RL on an FPGA amplifies the benefits of hardware sensing; the RL agent directly benefits from richer, faster data; and in turn, the success of such a system drives innovation in sensor precision and integration. This holistic approach (combining FPGA-based control with comprehensive sensing) ultimately leads to more intelligent, adaptive, and safe charging systems, aligning with the vision of smarter energy management through advanced sensor utilization [23,24].

Motivated by the above considerations, this work proposes a novel smart EV charging management system that integrates RL intelligence on an FPGA platform. The remainder of this article introduces the design and realization of the proposed system. Our proposed architecture is designed to manage multiple EV chargers, where each charger can operate at three levels: no current, low-level current, or high-level current. In addition, each charger can request priority charging, ensuring that urgent energy needs are met whenever possible. This flexible framework allows for effective load balancing and prioritization in dynamic environments. Importantly, the proposed approach is scalable as the same design principles can be applied to a system with a larger number of chargers, additional current levels, or multiple priority grades, ensuring adaptability for future infrastructure expansions. Based on the current literature on the hardware implementation of RL algorithms [20,25,26], it is feasible to effectively implement algorithms such as Q-learning [27] and SARSA (State Action Reward State Action) [28], which are tabular and discrete by definition.

First, we present a conceptual architecture based on Q-learning, detailing how the RL Agent perceives the environment and makes charging decisions to address grid stress and prioritization needs. We then describe the hardware implementation of this architecture on an FPGA, highlighting the techniques used to achieve efficient operation and real-time response. Finally, we demonstrate system performance through a series of simulations and hardware tests, showing how the FPGA-based RL controller improves charging results in various scenarios. This introduction sets the stage for the detailed development and results that follow, establishing the importance of intelligent RL-driven charging management and the benefits of deploying it on FPGA hardware. The subsequent sections will validate that such an approach can indeed meet the challenges of future EV charging networks, combining learning-based decision making with high-performance embedded processing.

## 2. Materials and Methods

This section is divided into two parts. The first part explains the Reinforcement Learning background, including its framework, the Q-learning algorithm, and the epsilon-greedy policy used in the system. The second part offers a detailed discussion of the hardware architecture, analyzes every functional subsystem, and discusses the design choices made.

### 2.1. Reinforcement Learning Background

**Figure 1:**
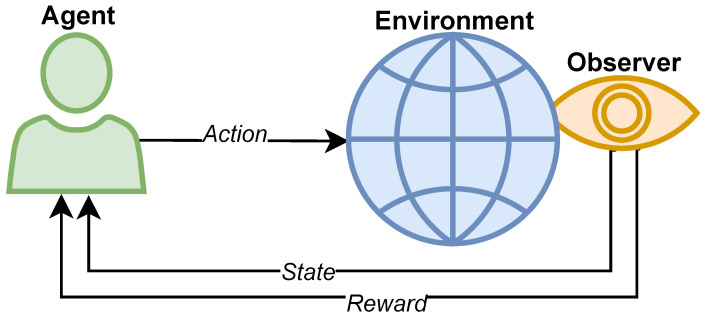
Reinforcement Learning typical framework.

A typical Reinforcement Learning framework is depicted in Figure 1.

Reinforcement Learning is a Machine Learning approach used to instruct an entity, known as an *Agent*, in executing a specific task by interfacing with its surrounding *Environment*.

The RL Agent modifies its behavior through a trial-and-error process facilitated by a Reinforcement (*Reward*) mechanism that serves as a measure of its overall task-solving ability [11]. The outcomes of the *Actions* performed by the Agent are collected and assessed by a component referred to as the *Interpreter*, which evaluates the new *State* of the Environment collecting data from the available sensors and allocates rewards to the Agent as appropriate.

The Interpreter can be either a separate entity or part of the Agent itself: in the latter scenario, the Agent and Interpreter combine to form a more sophisticated Agent capable of environmental perception and self-assessment. This is the kind of Agent applied in this work.

Among others, Q-learning is a prominent and frequently used Reinforcement Learning algorithm [29]. It is categorized under off-policy methods because it ensures convergence for any chosen policy of the agent.

It is worth mentioning that, during the initial stages of this research, we considered the option of developing our system using the Deep Q-learning or SARSA algorithms. The former was dismissed because of the significant resources needed to implement a CNN (Convolutional Neural Network) on an FPGA [19]. The latter, being an on-policy method, failed to reach adequate training convergence despite its feasible hardware architecture as per existing research. Consequently, we opted for Q-learning as a reliable option, known for its dependable convergence performance and efficient FPGA hardware implementation method [30].

The algorithm revolves around the use of a Quality Matrix or Q-Matrix. The dimensions of this matrix are N×Z, where *N* represents the number of possible states that the Agent can detect in the environment, and *Z* denotes the number of actions the Agent can take. Consequently, Q-learning operates within a discrete state-action space denoted as S×A. For a given state, represented by a row in the Q-Matrix, the optimal action is determined by identifying the maximum value in that row.

Initially, the Q-Matrix is populated with either random values or zeros at the start of training, and it is subsequently updated using the following:

(1) $$Q_{new}\left(s_{t},a_{t}\right)=\left(1-\alpha \right)Q\left(s_{t},a_{t}\right)+\;\alpha \left(r_{t+1}\;+\;\gamma \max_{a}Q(s_{t+1},a)\right)$$

where

- $s_{t}$ and $s_{t+1}$ are the present and the next state of the environment, respectively.
- $a_{t}$ and $a_{t+1}$ are the present and the next action, respectively, chosen by the agent and according to its policy.
- γ is the discount factor. Its value lies in the span [0,1], and it specifies the extent to which the agent must prioritize long-term rewards over immediate returns.
- α is the learning rate. Its value lies in the span [0,1], and it determines the level at which the most recent information should replace the existing knowledge.
- $r_{t+1}$ is the reward provided for taking the next action.

In [29], it has been shown that having the Q-Matrix is sufficient to derive the optimal Action Selection Policy (ASP) for an RL Agent [11].

An RL Agent is required to select its next Action. The manner in which this selection is executed is dependent upon its Action Selection Policy. Among various policies, the three most frequently utilized are listed below.

- *Random policy.* The Agent selects the subsequent Action at random without considering any information from the Q-Matrix. This strategy is typically employed during the initial phase of training.
- *Greedy policy.* The Agent consistently opts for the best Action based on the Q-Matrix. To determine the next Action, one must locate the highest value within the row corresponding to the present Environment State. This approach is typically utilized once the RL algorithm has converged to an optimal ASP.
- *ϵ-greedy policy.* This approach represents a blend of both Random and Greedy strategies. Based on a specified probability ϵ and an arbitrary probability *P*, the ASP is defined as random if P<ϵ, or greedy if P≥ϵ.
  Consequently, the Agent engages in a random Action with a probability of ϵ and opts for the optimal Action otherwise. This strategy is typically employed midway through the training process, allowing the Agent to maintain a balance between exploiting known options and exploring new potentials within the Environment.

### 2.2. System Hardware Architecture

**Figure 2:**
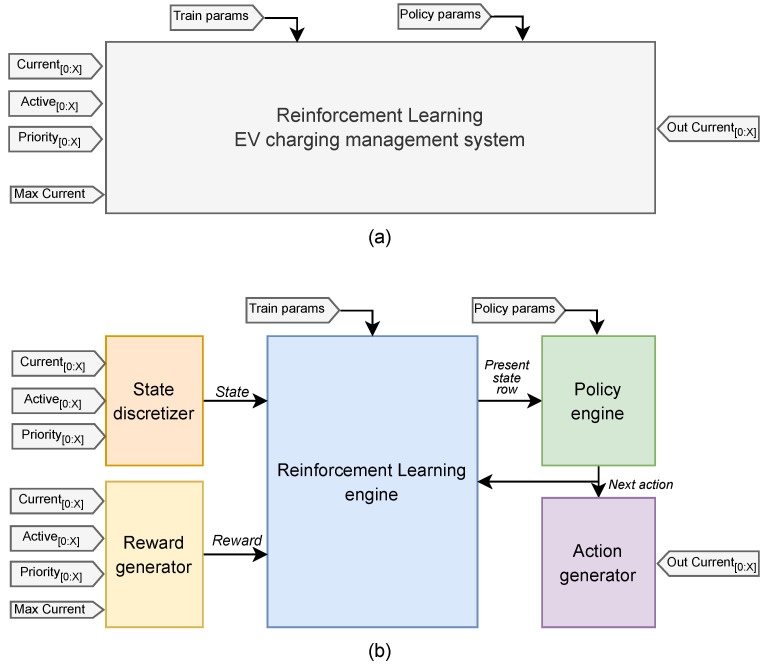
Top-level view of the EV charging management system: (**a**) general I/O view; (**b**) subsystems breakdown.

Based on the system specifications outlined in the Introduction, Figure 2 illustrates the proposed top-level hardware architecture.

Table 1 provides a comprehensive overview of the purpose and characteristics of each Input/Output signal.

The system is composed of different submodules, as follows:

- The *Reinforcement Learning engine* is responsible for maintaining the Q-Matrix, thus updating its values based on information received from other components of the system.
- The *Policy engine* is responsible for choosing the next action based on the Q-Matrix values associated with the present state of the environment.
- The *State discretizer* aggregates data collected by the sensors placed on the EV chargers to synthesize the state of the environment into a unique value, which is then entered into the reinforcement learning engine. This component is essentially part of the RL observer.
- The *Reward generator*, similar to the State discretizer, gathers data from the environment and outputs a reward value, assessing the agent’s actions. It serves as another part of the observer.
- The *Action generator* functions as a counterpart to the State discretizer. Based on the next Action determined by the Policy engine, which is a singular value, it produces the necessary current signals for deployment to each EV charger.

The following sub-sections provide detailed descriptions of each system component. Kindly note that vector signals are denoted by thicker lines accompanied by a slash (/).

#### 2.2.1. Reinforcement Learning Engine

This module processes the next state and the corresponding action derived from the environment observations and addresses the precise RAM and wordline to access the Q-Matrix values.

**Figure 3:**
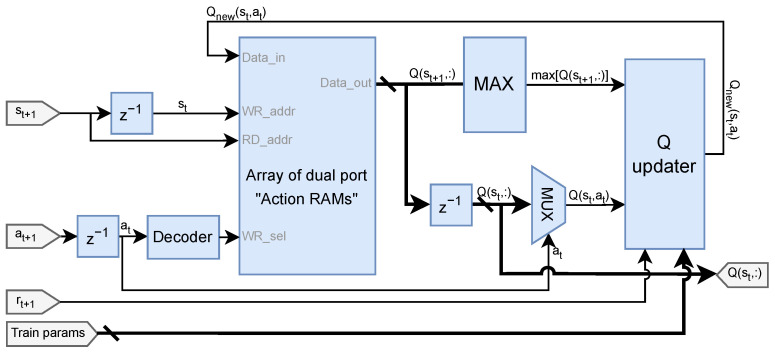
Hardware architecture of the Reinforcement Learning engine module.

The component is based on the research proposed by Spanò et al. [30], and its design is depicted in Figure 3.

The Q-Updater module executes the mathematical calculations outlined in Equation (1) to determine the updated value for the considered Q-Matrix element. This operation requires the highest value of the next state, the next reward from the observation, and the above covered training parameters.

Within our application, the Q-Matrix values and rewards are represented using the INT16 format. The states employ UINT9, Actions are coded with UINT5, and the parameters α and γ utilize 8-bit unsigned values, with only 1 bit designated for the integer part.

Further details about the working of the engine can be found in the referenced paper.

#### 2.2.2. Policy Engine

This module emulates the ϵ-greedy policy by selecting the next Action value from the range of possible Actions accessible to the Agent.

**Figure 4:**
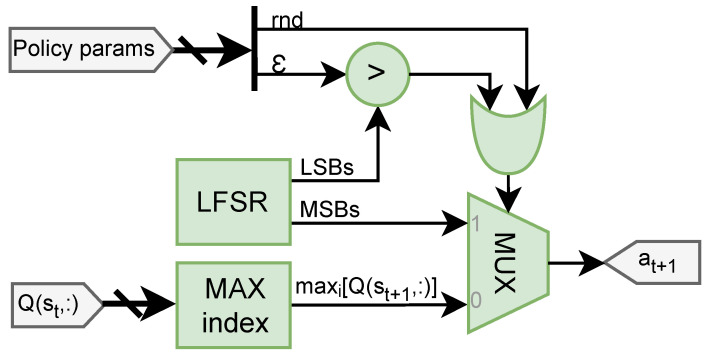
Hardware architecture of the Policy generator module.

The component is based on the research proposed by Cardarilli et al. [31], and its design is depicted in Figure 4.

A multiplexer (MUX) determines the selection between employing a greedy policy through input 0 and using a random approach through input 1.

The first involves choosing the index of highest value from the row in the Q-Matrix corresponding to the present state.

The latter involves creating pseudo-random numbers through a Linear Feedback Shift Register (LFSR). Specifically, the Most Significant Bits (MSBs) are utilized to produce a random action value, whereas the Least Significant Bits (LSBs) serve as a comparison metric for the parameter ϵ, which is the probability *P* of the policy. Essentially, a random output will be generated if ϵ>P or the rnd parameter is enforced.

Further details about the working of the generator can be found in the referenced paper.

#### 2.2.3. State Discretizer

To implement the Q-learning algorithm that operates within a discrete state space, each electric vehicle status from the sensors placed on the chargers must be encoded into a distinct value that represents every possible combination of charger conditions.

Every charger supplies the EV management system with three signals: A, P, and C.

- *A* serves as the Active Boolean signal to show whether a vehicle is connected to the charger.
- *P* represents the Priority Boolean indicator, which signals whether the vehicle is requesting Priority in the waiting line.
- *C* denotes the Current provided by the charger. In our configuration, it can take the values [0, 1, 2], which correspond to no current and two progressively higher levels.

Based on the considerations mentioned, a single charger can be in 7 different conditions, as shown in Table 2.

Thus, each charger is encoded by an integer *B* ranging from 0 to 6.

Considering that every possible combination of charger conditions must be taken into account and each must be assigned a distinct state, we propose a state encoding derived from the conversion of the base-7 numeric system. Indeed, each charger corresponds to a digit *B* in a base-7 system. Consequently, for X−1 chargers, the discretization of the states can be performed as described below:

(2) $$s=B_{X}\;\cdot \;7^{X}+B_{X-1}\;\cdot \;7^{\left(X-1\right)}+...+B_{1}\;\cdot \;7+B_{0}$$

**Figure 5:**
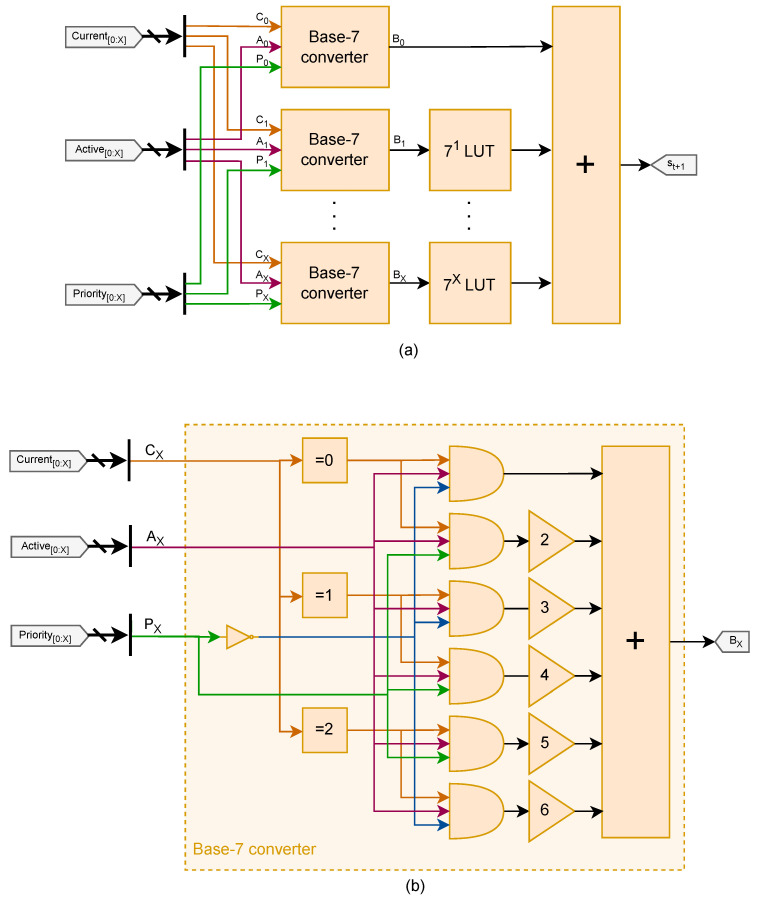
(**a**) Hardware architecture of the State discretizer module; (**b**) detail of the base-7 converter submodule.

Figure 5 illustrates the architecture of the State discretizer that executes Equation (2).

The base-7 converter, as illustrated in Figure 5b, calculates the *B* digits of the chargers by checking all the conditions of Table 2 and then implementing a constant gain accordingly. The multiplications by powers of 7 utilizes precomputed LookUp Tables (LUTs). All partial results are then added to derive an appropriate state value for the Q-learning engine.

The architecture just described is conceptual, as each operation in Figure 5a is typically translated into a LUT configuration during a standard FPGA synthesis process.

The total number of states for the RL algorithm, considering 7 possible configurations per charger and X−1 charging points, can be easily expressed as

(3) $$N_{s}=7^{X}$$

In the following sections, we prove the effectiveness of our method using an experimental setup with 3 chargers, leading to a total of $N_{s}=343$ states. However, this is applicable to any other system configuration and can be tailored to meet the designer’s requirements.

#### 2.2.4. Action Generator

Similar to before, the Q-learning algorithm operates within a discrete Action space, which means that the specific Action value produced by the Policy engine must be translated into a current level of [0, 1, 2] for each charger.

As for the State discretizer, we used a base conversion approach, in this case a base-3 one. An example of the mapping between Action and current level for a 3-charger setup is presented in Table 3.

It can be seen that it executes the reverse operation of

(4) $$a=C_{X}\;\cdot \;3^{X}+C_{X-1}\;\cdot \;3^{\left(X-1\right)}+...+C_{1}\;\cdot \;3+C_{0}$$

which is the same approach of Equation (2).

**Figure 6:**
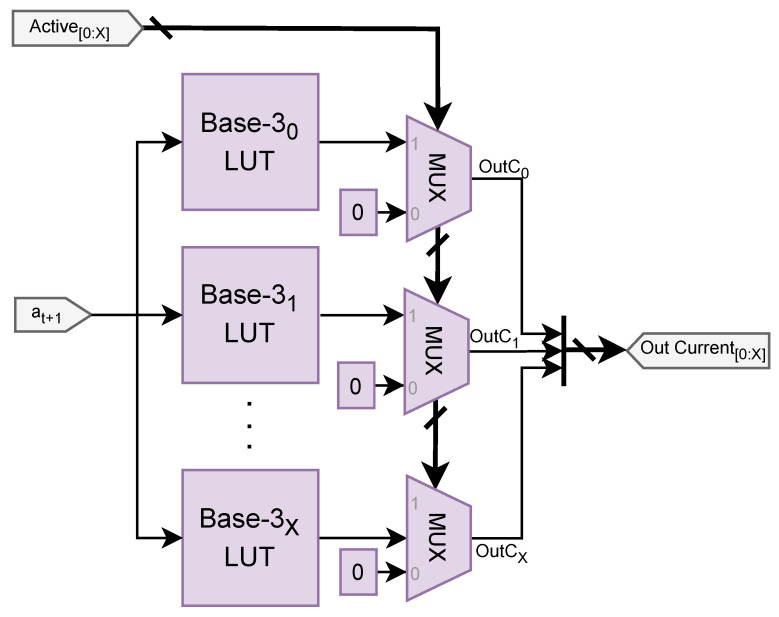
Hardware architecture of the Action generator module.

Figure 6 illustrates the architecture of the Action generator module.

Every base-3 LUT includes the digits for the conversion from Action to current level (as seen in Table 3). A series of MUXes is configured to deactivate the current for any charger that does not have a high active signal, ensuring that no current is supplied when no vehicle is attached to the charger, and to avoid any misinterpretation with the sensor data.

The total number of actions for the RL algorithm, considering 3 possible levels of current per charger and X−1 charging points, can be easily expressed as

(5) $$N_{a}=3^{X}$$

In the following sections, we prove the effectiveness of our method using an experimental setup with 3 chargers, leading to a total of $N_{a}=27$ actions. However, this is applicable to any other system configuration and can be tailored to meet the designer’s requirements. If we match these data to the number of states in the considered configuration Equation (3), we achieve a Q-Matrix size of

(6) $$N_{s}\times N_{a}=343\times 27$$

#### 2.2.5. Reward Generator

To train the RL management system, it is necessary to provide a reward to the Q-learning engine based on the observation of the environment. The goals of the system are the following:

- *Prevent overcurrent.* The aggregated current output of the chargers ($Agg_{C}$) should not exceed the specified limit; in our tests, we used a maximum of 4 current units (MaxCurrent).
- *Deploy the maximum accessible current.* Power must be distributed evenly among all active vehicles.
- *Mechanism for prioritization.* Vehicles that need priority in the waiting line must be allocated the maximum available current, whenever feasible.
- *Ensuring Quality of Service (QoS).* Every active vehicle must obtain a minimum current supply, even when there are other vehicles with higher priority.

To achieve this and to provide a reward *R*, the Reward generator module evaluates the charger conditions (as outlined in Table 2) and assigns a Partial Reward PR to each. These initial values are then summed to produce the Cumulative Reward CR. However, if an overcurrent occurrence is identified, CR must be ignored, and instead, a significantly negative reward is given. The rewarding values are shown in Table 4.

The reward values were defined by analyzing several training trials, leading to the following general rules:

- When there is no vehicle connected and the system yields no current (0a_0c), a modest positive reward should be assigned. The same amount should be allocated when a minimal current is supplied to a low-priority vehicle (1c_lp). These scenarios serve as base cases.
- When a low-priority vehicle is fed a higher current, the reward has to be increased ×2 (2c_lp).
- A high-priority vehicle receiving current has to be rewarded from ×2.5 (2c_hp) to ×3 (1c_hp) with respect to a low-priority one.
- When a vehicle is connected yet not receiving any current, it should incur a penalty consisting of a negative reward proportional to that awarded when the maximum current is supplied (0c_lp, 0c_hp).
- If any overcurrent is detected, the reward has to be ×2 the one in case of a high-priority vehicle not receiving any current (ov_c).

The earlier heuristics remain effective as long as their proportions are preserved. Nonetheless, please be aware that adjusting the learning rate α might be necessary to achieve an appropriate convergence speed. Additionally, the suggested guidelines can be proportionally expanded if the system provides more current levels.

**Figure 7:**
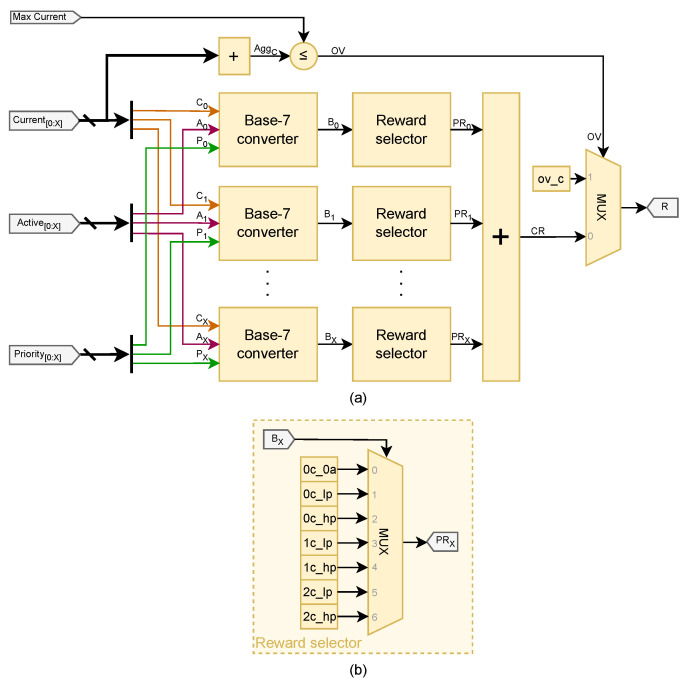
(**a**) Hardware architecture of the Reward generator module; (**b**) detail of the Reward selector submodule.

The rewarding mechanism can be summarized as

(7) $$R=\left\{\begin{array}{cc} CR=PR_{X}+PR_{X-1}+...+PR_{1}+PR_{0}, & \mathrm{if}\;Max\;Current\leq Agg_{C}\\ ov_{c}, & \mathrm{otherwise} \end{array}\right.$$

and can be easily understood by looking at the architecture illustrated in Figure 7.

The base-7 converters match those used in the State discretizer module, as shown in Figure 5b. As a result, they are merged during the FPGA synthesis process. Figure 7b illustrates that the Reward selector submodules determine the appropriate Partial Rewards by evaluating the condition of the chargers. Subsequently, a Cumulative Reward is calculated using an adder.

Eventually, a MUX selects the appropriate overcurrent reward after comparing the cumulative deployed current with the module’s maximum current input.

As stated above, the architecture just described is conceptual, as each operation in Figure 7a is typically translated into a LUT configuration during a standard FPGA synthesis process.

## 3. Results

This section is split into two distinct parts. The first part details the experiments conducted to evaluate the correct functional performance of the system in both simulation and actual hardware platforms. The second part provides all the details regarding the implementation results on the chosen target FPGA hardware, specifically the AMD ZCU104 development board, which features the Zynq UltraScale+ XCZU7EV-2FFVC1156 MPSoC.

### 3.1. Functional Verification

The experimental setup includes three chargers. The training was carried out using the following parameters:

- No. of epochs: 100;
- N0. of iterations per epoch: 2560;
- Learning rate α=1;
- Discount factor γ=0.015625.

The Policy engine was configured through an ϵ-greedy strategy employing the following parameters:

- Initial ϵ=0.875;
- Decrement after each epoch: Δϵ=−0.046875;
- Minimum ϵ=0.015625.

Upon selecting a suitable set of rewards, we carried out 50 training experiments. In each instance, the system reliably exhibited the desired behavior, resulting in closely aligned values in the final Q-Table.

**Figure 8:**
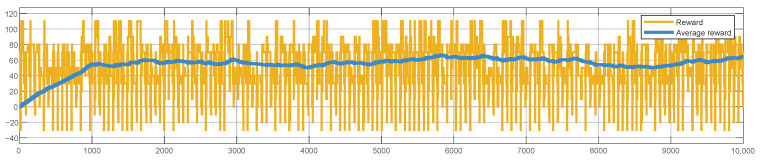
System simulation. Results: reward values over iterations.

The convergence of the RL algorithm is evident from the reward values depicted in Figure 8, which shows the system operating in inference mode.

It is important to note that, in all the next plots, the *x*-axis denotes the clock cycles.

We evaluated the average reward using moving windows of 1000 samples. The value is estimated to be around 60, which is consistent with the reward values chosen in Table 4.

The data used for both the training and testing phases of the system are inspired from the ACN-Data—a Public EV Charging Dataset from CalTech University [32]. We were unable to use the CalTech dataset or other existing datasets as they are because they are designed to work with much more complex algorithms and on significantly more energy-hungry machines meant for cloud applications. For this reason, we redefined the problem by considering quantized levels and real-time provided charging priorities. The entire system was designed to achieve extremely low latency in order to operate in real time while preserving realistic data collected by the sensors on the chargers.

#### 3.1.1. Simulation Results

Upon completing the training, the performance of the management system was evaluated through simulation in a Simulink environment. Various random inputs were introduced to mimic the vehicles that connect to the chargers along with the current data collected by the sensors, some of which might request prioritized charging. Our objective is to verify that all the established goals have been met.

**Figure 9:**
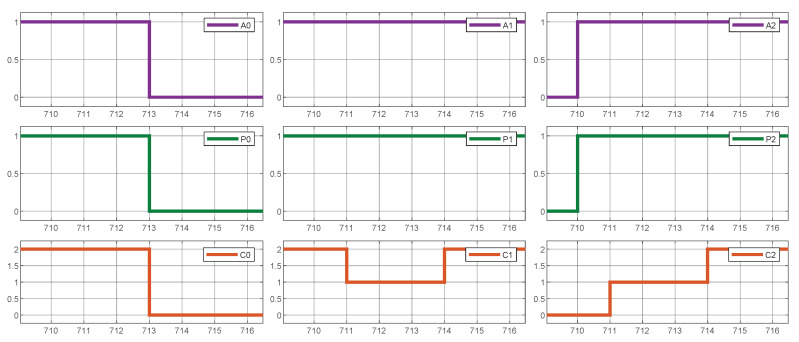
System simulation results. Overcurrent avoidance.

As illustrated in Figure 9, the aggregated current of the chargers ($Agg_{C}$) always remains within the designated threshold of 4 current units; in fact, the sum of C0+C1+C2 always reaches that value.

**Figure 10:**
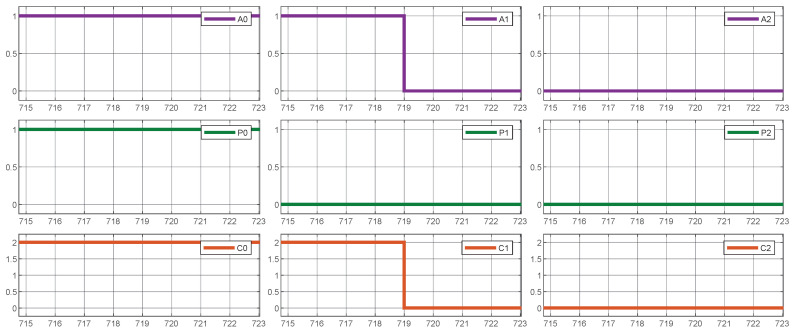
System simulation result. Maximum current always deployed.

As shown in Figure 10, the system consistently utilizes the maximum available current, distributing it evenly across all active vehicles.

**Figure 11:**
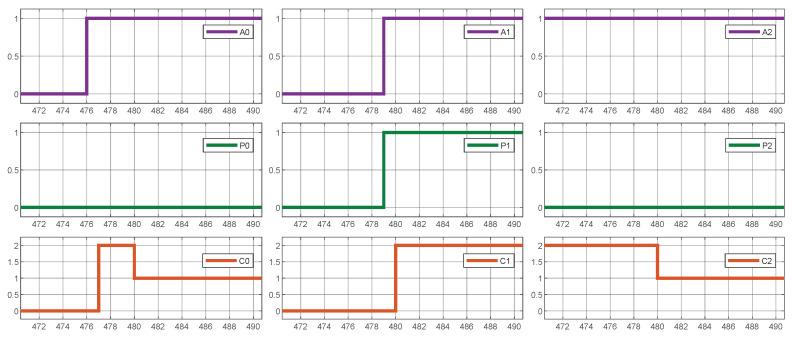
System simulation results. Prioritization mechanism.

Figure 11 illustrates the proper functioning of the prioritization mechanism, demonstrating how vehicles requiring priority on the waiting line receive the maximum available current whenever it is practicable. This phenomenon is noticeable at time 480, where the vehicle connected to charger 1 demands priority, causing a reduction in the current supplied to other low-priority vehicles to meet the demand.

**Figure 12:**
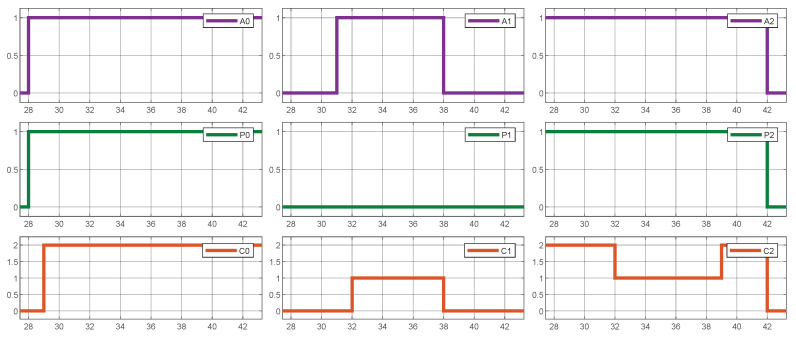
System simulation results. Quality of Service (QoS) assurance.

In conclusion, Figure 12 demonstrates how Quality of Service is maintained by guaranteeing that every active vehicle obtains a minimum current supply, regardless of the presence of vehicles with higher priorities. In the given example, as evident in the periods 32 to 38, low-priority vehicle 1 is supplied with a minimum amount of current, which temporarily reduces the current allocated to high-priority vehicle 2.

#### 3.1.2. Hardware Deployment Results

The architecture under discussion has been designed at the Register Transfer Level (RTL) utilizing VHDL. The implementation is presented on the specified ZCU104 FPGA hardware by seamlessly integrating the Simulink and Vivado tools. Furthermore, the architectural design has been validated through testing with the MathWorks FPGA-in-the-Loop technique (FIL) [33].

**Figure 13:**
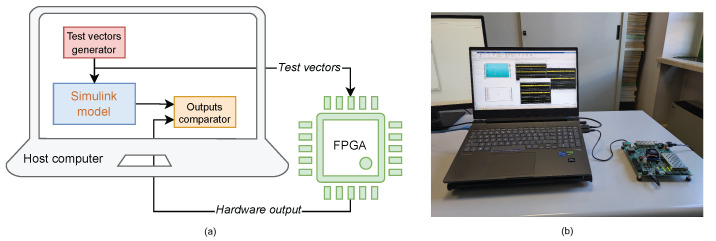
FPGA in-the-Loop (FIL) testing framework: (**a**) FIL workflow principle; (**b**) FIL real hardware setup.

FIL is a design verification procedure provided by MathWorks, which allows a direct real-time link between a design implemented on an FPGA and a MATLAB/Simulink simulation environment. This method supports co-simulation by comparing test signals from both the Simulink model and the actual FPGA hardware, guaranteeing that everything functions as expected. Figure 13 demonstrates this process.

**Figure 14:**
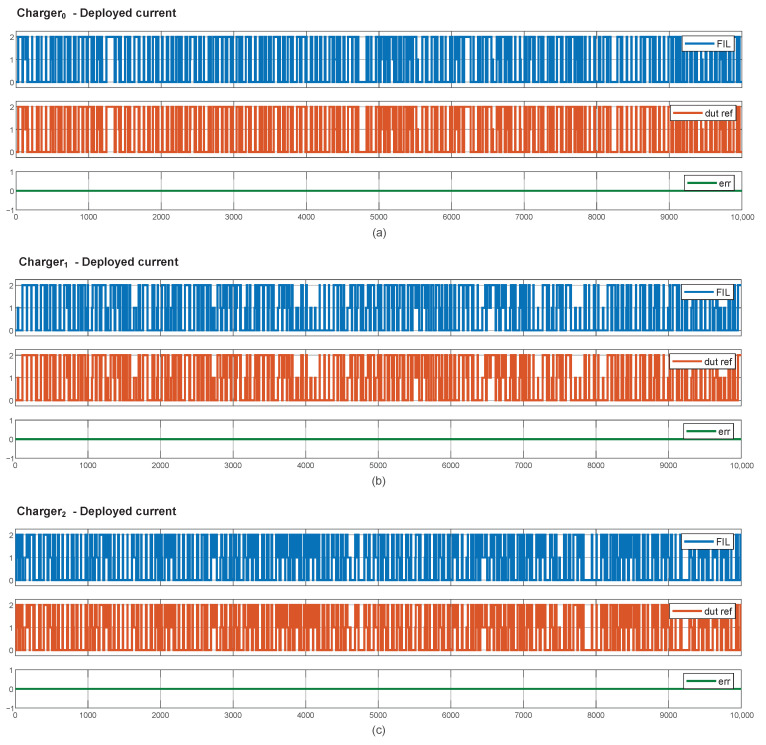
Hardware deployment results. Comparison between the deployed current by the FPGA (FIL), by the simulation model (dut ref), and the difference between them (err): (**a**) Charger 0, (**b**) Charger 1, and (**c**) Charger 2.

Figure 14 presents the FIL results, illustrating that the current readings from the sensors placed on the three chargers in both the simulation and the actual hardware match perfectly. With the same inputs, injected by the JTAG interface, the outputs remain consistently the same, resulting in the absence of errors.

This shows that the hardware deployment has been successfully achieved.

### 3.2. Hardware Implementation

**Figure 15:**
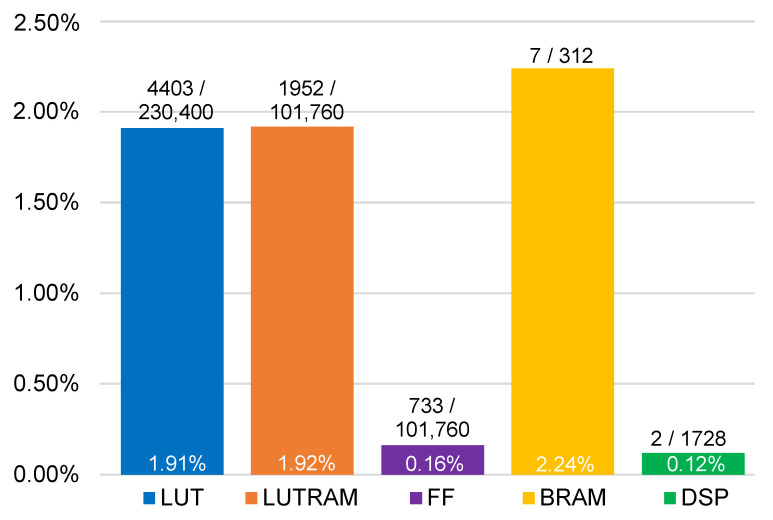
Hardware resources of the system.

The RTL was synthesized and implemented on the ZCU104 development platform using Vivado 2024.2; the resources requirements are shown in Figure 15. For each resource, the utilized quantity is displayed atop the bars alongside the total count, while the corresponding percentage is shown within the bars.

As evidenced, the resource consumption is minimal, indicating the design’s high efficiency and possibility to scale with increasingly complex scenarios and functions.

**Figure 16:**
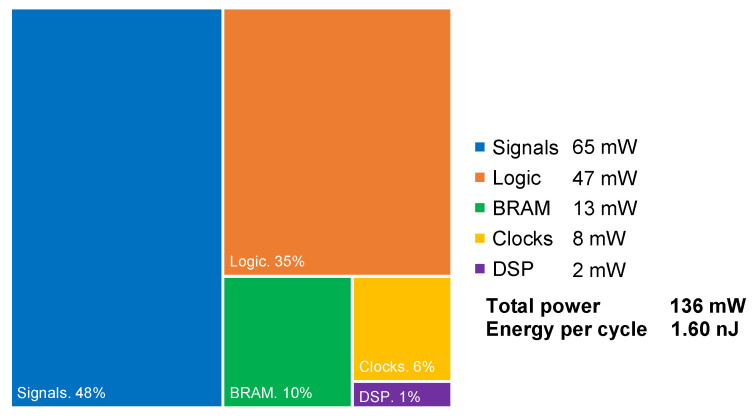
System dynamic power breakdown and energy per cycle. Clock at 84.746 MHz.

Figure 16 illustrates the breakdown of dynamic power dissipation, assessed using a worst-case vectorless approach at the highest achievable clock frequency of 84.746 MHz. Note that, due to the system’s efficient architecture, it provides valid current signals with every clock cycle, making it ideal for real-time applications. The system can adjust to sudden changes in the surrounding environment, as demonstrated in the Functional Verification section, by modifying the deployed current with a consistent latency of 34.3 ns.

The energy consumption and power dissipation of the management system are minimal, allowing for its implementation in embedded applications and possibly enabling the use of an energy harvesting power source, like a small solar panel.

Unfortunately, it is difficult to compare our FPGA implementation with CPU or GPU ones. In fact, although different works in the literature use Reinforcement Learning to manage electric vehicle charging, they focus mainly on the algorithmic aspect, providing little or no information on latency, energy efficiency, and other related metrics [14,15,16,17,18].

#### Scalability Considerations

The results of the implementation of the examined use case, along with the data from the literature [30], clearly demonstrate that the proposed architecture is quite light. If additional chargers, current levels, or prioritization orders need incorporation, the primary concern of the designer becomes the memory needed to store the Q-Table values. In fact, the RAM needs can be outlined based on the number of states $N_{s}$, the number of actions $N_{s}$, and the bits *n* used to represent the values.

(8) $$MEM_{Q}=N_{s}\times N_{s}\times n\;\;\mathrm{bits}$$

In the tests under consideration, we needed 18.522 KB. For instance, increasing the number of chargers to 5, while keeping all other factors constant, leads to a RAM demand exceeding 8.168 MB, surpassing the ZCU104 platform’s available internal RAM of 4.75 MB.

With the exponential rise in the number of states and actions, utilizing external DRAM can effectively tackle the problem without having to limit the Q-Table to integrated FPGA BRAMs, thereby not constraining the proposed approach. The architecture was conceived with this consideration in mind.

## 4. Discussion

This study has shown the effective deployment of a conceptual framework aimed at intelligently managing electric vehicle charging systems through Reinforcement Learning on FPGA platforms. The system introduced has been efficient, fast, and economical in terms of resources while still ensuring strong performance. A critical enabler of this architecture is the integration of hardware-based sensing technology, which provides the environmental feedback essential for real-time decision making. The sensors embedded in the charging infrastructure, monitoring variables such as current, voltage, and priority status, supply the RL agent with the information required to perceive grid and vehicle states. The experimental results validate that the architecture can handle multiple EV chargers, optimize current distribution, prioritize tasks, and maintain service quality.

The key findings reveal that the proposed system achieved the convergence of training, highlighting its ability to both learn effectively and execute optimal policies. The system adeptly avoided overcurrent scenarios while consistently distributing the maximum current allowed to active chargers. Furthermore, the prioritization mechanism efficiently directed the priority charging to high-demand vehicles without diminishing overall performance.

From a hardware perspective, the implementation of three chargers on the ZCU104 FPGA development board achieved notable efficiency with minimal resource consumption. The light architecture of this system and efficient energy use make it ideal for resource-limited embedded applications. Furthermore, scalability factors demonstrate that the proposed design can seamlessly expand to larger implementations by adhering to the same architectural guidelines.

Future studies might investigate the fusion of advanced deep Reinforcement Learning methodologies to handle larger and more intricate state-action domains. Additionally, leveraging external memory or hardware-software co-designs might help address resource limitations. Incorporating real-time data, e.g., fluctuating electricity prices, renewable energy supply, or preferences of vehicle users, could further refine decision processes. These prospects, combined with the system’s low energy usage and optimized architecture, set the stage for implementing intelligent, embedded EV charging managers within practical smart grid setups.

## Figures and Tables

**Table 1:** EV charging system I/O signals map and related details.

Port Name	I/O	Type	Description
$Current_{\left[0:X\right]}$	I	Vector	Current deployed by each EV charger
$Active_{\left[0:X\right]}$	I	Vector	Boolean indication if a vehicle is connected to the charger
$Priority_{\left[0:X\right]}$	I	Vector	Boolean indication if the vehicle requires priority charging
MaxCurrent	I	Scalar	Maximum overall current that can be deployed by all chargers simultaneously
Trainparams	I	Vector	Collection of parameters required for the training of the RL algorithm
- α	I	Scalar	Learning rate
- γ	I	Scalar	Discount factor
- train	I	Scalar	Boolean signal to enable the training of the algorithm
Policyparams	I	Vector	Collection of parameters required for generating the next action
- rnd	I	Scalar	Boolean signal to enable a full random policy
- ϵ	I	Scalar	ϵ-greedy policy value
$Out\;Current_{\left[0:X\right]}$	O	Vector	Current that has to be deployed by each EV charger

**Table 2:** State encoding for each EV charger.

A	P	C	Charger Condition	Notes
**0**	**0**	**0**	0	Not active
**1**	**0**	**0**	1	Active, no current, low priority
**1**	**1**	**0**	2	Active, no current, high priority
**1**	**0**	**1**	3	Active, current level 1, low priority
**1**	**1**	**1**	4	Active, current level 1, high priority
**1**	**0**	**2**	5	Active, current level 2, low priority
**1**	**1**	**2**	6	Active, current level 2, high priority

**Table 3:** Example of action mapping for a 3-charger setup.

Action	Current 2	Current 1	Current 0
**0**	0	0	0
**1**	0	0	1
**2**	0	0	2
**3**	0	1	0
**4**	0	1	1
**5**	0	1	2
**6**	0	2	0
**…**	…	…	…
**25**	2	2	1
**26**	2	2	2

**Table 4:** Reward values and their related names in the architecture.

Charger State	Reward Value	Reward Name	Notes
**0**	+10	0a_0c	Not active
**1**	−20	0c_lp	Active, no current, low priority
**2**	−50	0c_hp	Active, no current, high priority
**3**	+10	1c_lp	Active, current level 1, low priority
**4**	+30	1c_hp	Active, current level 1, high priority
**5**	+20	2c_lp	Active, current level 2, low priority
**6**	+50	2c_hp	Active, current level 2, high priority
**OV**	−100	ov_c	Overcurrent detected

## Data Availability

Dataset available on request from the authors.

## Abbreviations

The following abbreviations are used in this manuscript:

EV	electric vehicles
RL	Reinforcement Learning
FPGA	Field-Programmable Gate Arrays
GPU	Graphics Processing Unit
CPU	Central Processing Unit
SARSA	State Action Reward State Action
CNN	Convolutional Neural Network
LFSR	Linear Feedback Shift Register
MSB	Most Significant Bit
LSB	Least Significant Bit
RTL	Register Transfer Level
VHDL	VHSIC Hardware Description Language
VHSIC	Very High Speed Integrated Circuits
FIL	FPGA-in-the-Loop
